# Data-driven selection of conference speakers based on scientific impact to achieve gender parity

**DOI:** 10.1371/journal.pone.0220481

**Published:** 2019-07-31

**Authors:** Ann-Maree Vallence, Mark R. Hinder, Hakuei Fujiyama

**Affiliations:** 1 Discipline of Psychology, College of Science, Health, Engineering, and Education, Murdoch University, Perth, Australia; 2 School of Medicine, College of Health and Medicine, University of Tasmania, Hobart, Tasmania, Australia; San Francisco State University, UNITED STATES

## Abstract

A lack of diversity limits progression of science. Thus, there is an urgent demand in science and the wider community for approaches that increase diversity, including gender diversity. We developed a novel, data-driven approach to conference speaker selection that identifies potential speakers based on scientific impact metrics that are frequently used by researchers, hiring committees, and funding bodies, to convincingly demonstrate parity in the quality of peer-reviewed science between men and women. The approach enables high quality conference programs without gender disparity, as well as generating a positive spiral for increased diversity more broadly in STEM.

## Introduction

Demand in the science community for approaches to ensure diversity and inclusion is growing [1–5]. Gender disparity in academia has been acknowledged for some time. In science, technology, engineering, and mathematics (STEM), women represent approximately half of PhD graduates since 1990s but only approximately a quarter of professors [6,7]. Although calls for approaches to help achieve gender parity in STEM have been numerous, progress is slow.

Recently, gender disparity in invited speaker presentations at scientific conferences has attracted much attention, with evidence of such disparity in STEM conference programs including (but not limited to) fields such as sport and exercise medicine [8], evolutionary biology [9], mathematics [10], ecology [11], geophysical sciences [12], and microbiology [13]. In the field of neuroscience, BiasWatchNeuro has published gender data of speakers to increase accountability for gender disparity in conference programs; data extracted from BiasWatchNeuro (on 12/20/18) indicated that only 27% of invited speakers across ~400 neuroscience conferences (between 2014–2018) were women [14]. Although some neuroscience conferences are attaining, or exceeding, parity in invited speakers, more than 80% of conferences had less than 50% women in their invited speaker programs [14]. Given that such opportunities are critical for career development, approaches to achieve gender parity in conference programs while maintaining the high scientific standards expected in conferences programs are needed. The traditional approach to speaker selection is based on who the organizing committee knows, or whose work they are familiar due to overlap with their own research disciplines. Therefore, a data-driven approach to provide credibility to speaker selection is critically needed. The development of such approaches that maximize objectivity is particularly important because the lack of diversity of speakers at scientific conferences is not only detrimental to individual careers: growing evidence demonstrates the positive effect of diversity within teams on the progression of science [1,5,15–18].

In recent years, a number of neuroscience societies have developed and implemented equity and diversity policies that can guide the composition of conference programs [19] (e.g. Australasian Cognitive Neuroscience Society https://www.acns.org.au/wp-content/uploads/2018/08/ACNS_Equity_Diversity_Policy_Final_Nov2016.pdf). In addition, equitable gender representation can be estimated by online calculators or achieved by following explicit guidelines for conference committees [20]. The development and uptake of these approaches provide evidence of a willingness to compose more equitable conferences and improve the conventional subjective speaker selection process. However, none of the currently available approaches provide information regarding *how* to identify conference speakers to invite based on scientific quality. The subjective nature of speaker selection decisions likely plays a large role in the persistent gender disparity in neuroscience conference programs. Here, we developed a world first, data-driven approach to speaker selection to directly address this issue.

Our two-step approach to invited speaker selection is aimed at achieving high quality, gender balanced, conference programs. First, we audited the top ten neuroscience journals (indexed by SCImago Journal and Country Rank; SJR), (i) ranking publications from 2012–2016 by citation count, as well as identifying (ii) gender, (iii) field-weighted citation impact (FWCI), and (iv) total publication count of the first and last authors. Second, we used these data to establish a database of authors who have published high quality, peer-review science from which potential speakers could be selected for conferences. Identifying potential speakers based on these metrics—which are frequently used by researchers, hiring committees, and funding bodies—can provide convincing evidence of parity in the *quality* of peer-reviewed science between men and women at the highest level. This innovative approach extends beyond the currently available tools by identifying particular individuals as potential speakers based on their recent high quality peer-reviewed science. Notably, this approach can have an immediate effect to improve the representation of women invited speakers at neuroscience conferences, and will likely have a medium-to-long-term effect to improve the progression of women scientists to senior levels within STEM. Furthermore, this will enable science from broader perspectives to be presented at scientific conferences, which will improve the quality of the science presented.

## Database construction

**Fig 1:**
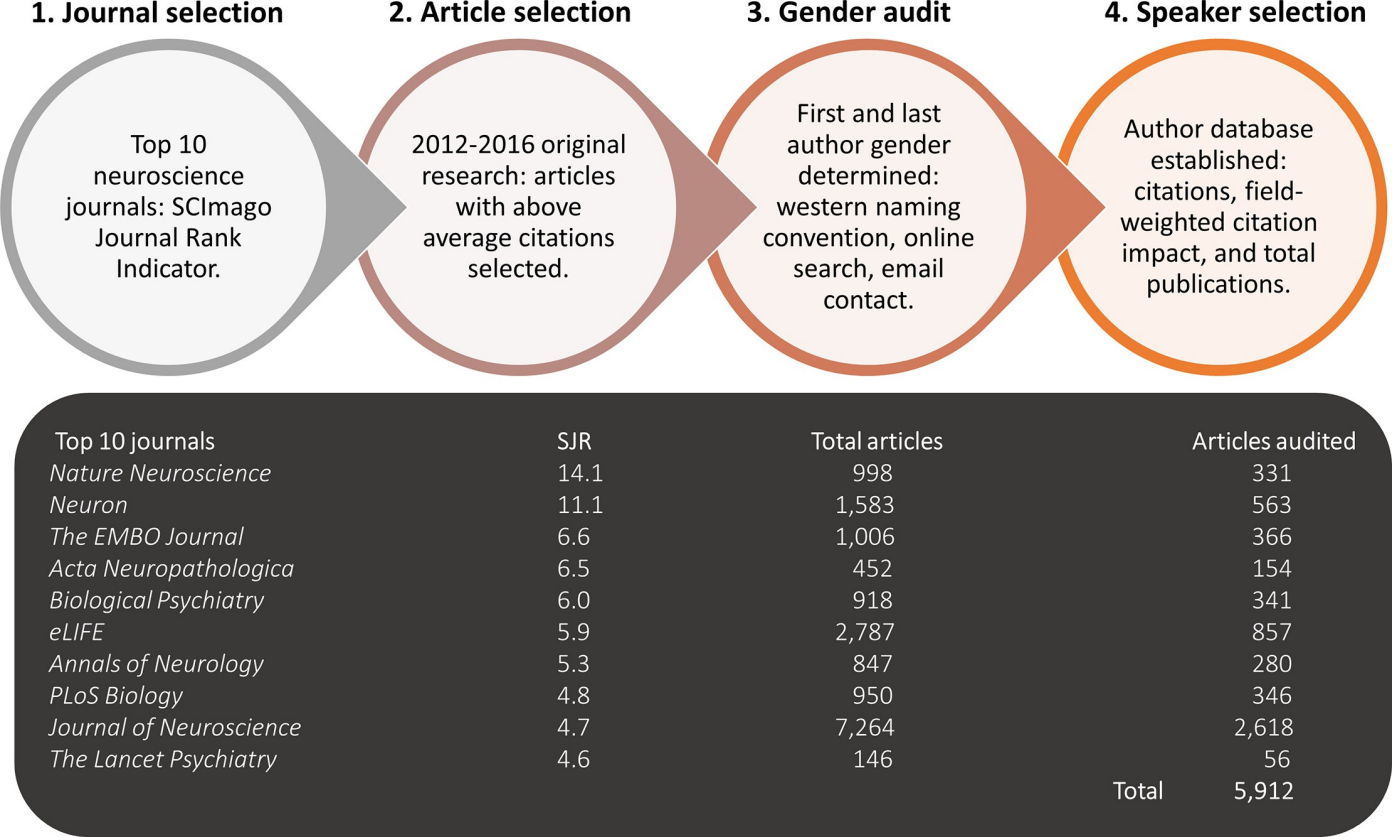
Selection procedure for creating a database for speaker selection.

The study was approved by the Murdoch University Human Research Ethics Committee (2017/206). Fig 1 shows the study procedure (journal ranking data and citation reports.

### Journal selection

Neuroscience journals were ranked using the SJR indicator system and Web of Science using the filters ‘neuroscience’ as subject and ‘journal’ as publication type (using ‘all subject categories’ and ‘all regions/countries’). (SJR is a publicly available portal that provides scientific bibliometric indicators for journals and countries based on information contained in the Scopus database; SJR takes into account the number of citations received by the journal as well as the prestige of the citing journals [21]). The top ten journals comprising ≥50% original research articles were selected for auditing (see Fig 1). (Note: *Molecular Psychiatry* was excluded because more than 60% of publications reported authors’ initials only, making gender determination very difficult).

### Article selection

Thomson Reuters’ Web of Science database was used to retrieve all articles published in the top 10 neuroscience journals from 2012–2016. The journal title was entered in the ‘publication name’ search field, and results were restricted to ‘original articles’ with retractions excluded. Web of Science was used to generate citation reports for each of the five years for each of the target journals. (As Web of Science frequently updates information about citation data, all reports were generated and downloaded on the same day to maintain internal validity.) From these reports, total citations and average citations per year were calculated for each original research article in the selected journals (citations from 2012–2016 for all journals except *Lancet Psychiatry*, for which citation data were only available from 2014–2016). Articles were selected for the author gender audit if their total citation count was greater than the average total citations for the journal in which the article was published in the year of publication. Therefore, for each journal, five cut-off points were determined: one for each publication year (except *Lancet Psychiatry*, which only had data from 2014–2016). Histograms showing the total number of original research articles, citations, and the cut-off points for each journal for each year are provided in the supporting information (S1–S10 Figs). In total, 5,912 original research articles were audited; the names of the first and last authors were extracted from the citation reports for these research articles (with the authors of single-author publications being classified as first author).

### Gender identification

The gender of first and last authors of the selected articles was determined to be a man, woman, or unknown. Whilst there is some deviation, in many fields (including neurosciences), the first author is considered the lead author who conducted the research: most often the first author is an early-career researcher (commonly defined as PhD student to 5 years post-PhD) or mid-career researcher (commonly defined as 5–15 years post-PhD) [22–24]. The last author is considered the senior author whose supervision, mentorship, and expertise make a significant intellectual contrtibution to the research, and who takes on the takes on the responsibility for the research outcomes [22–24].

Gender determination (using western naming convention) was completed independently by two investigators, and then cross-referenced. If gender could not be determined using this method, or the name was indeterminate or androgynous, an electronic search was conducted using institutional and academic networking websites: gender was determined if the online resources included the author’s name, photo (with clear gender identification) and either a reference to the article or the author’s affiliation (listed in the article). If gender of first or senior authors could not be determined using either of these methods (6.9%), the corresponding author was emailed to request gender identity information (email response rate: 20%). In total, we attempted to determine the gender of 11,791 authors (5,912 first authors and 5,879 senior authors; discrepancy in first and senior author numbers due to single author publications): the gender of 655 authors (5.6%) could not be determined.

### Quality metrics

The weighted total citations (2012–2016) were obtained by dividing the total citation counts for each paper by the number of years since its publication. The FWCI (2012–2016) and their total number of career publications were obtained for the top 100 first and last authors from SciVal. (Note: two first authors did not have an identifiable FWCI using SciVal. These two authors were excluded from the first author database and the next ranked authors were included (i.e. authors ranked 101 and 102). All last authors had an identifiable FWCI.)

## Research impact of potential speakers

The 100 articles with the highest weighted total citations were used to create a database of potential speakers, including lists for first author and last author. The rank order within these lists was then adjusted based on FWCI (all of the data for the top-100 first and senior authors are available as supporting information: ‘speaker database’).

**Fig 2:**
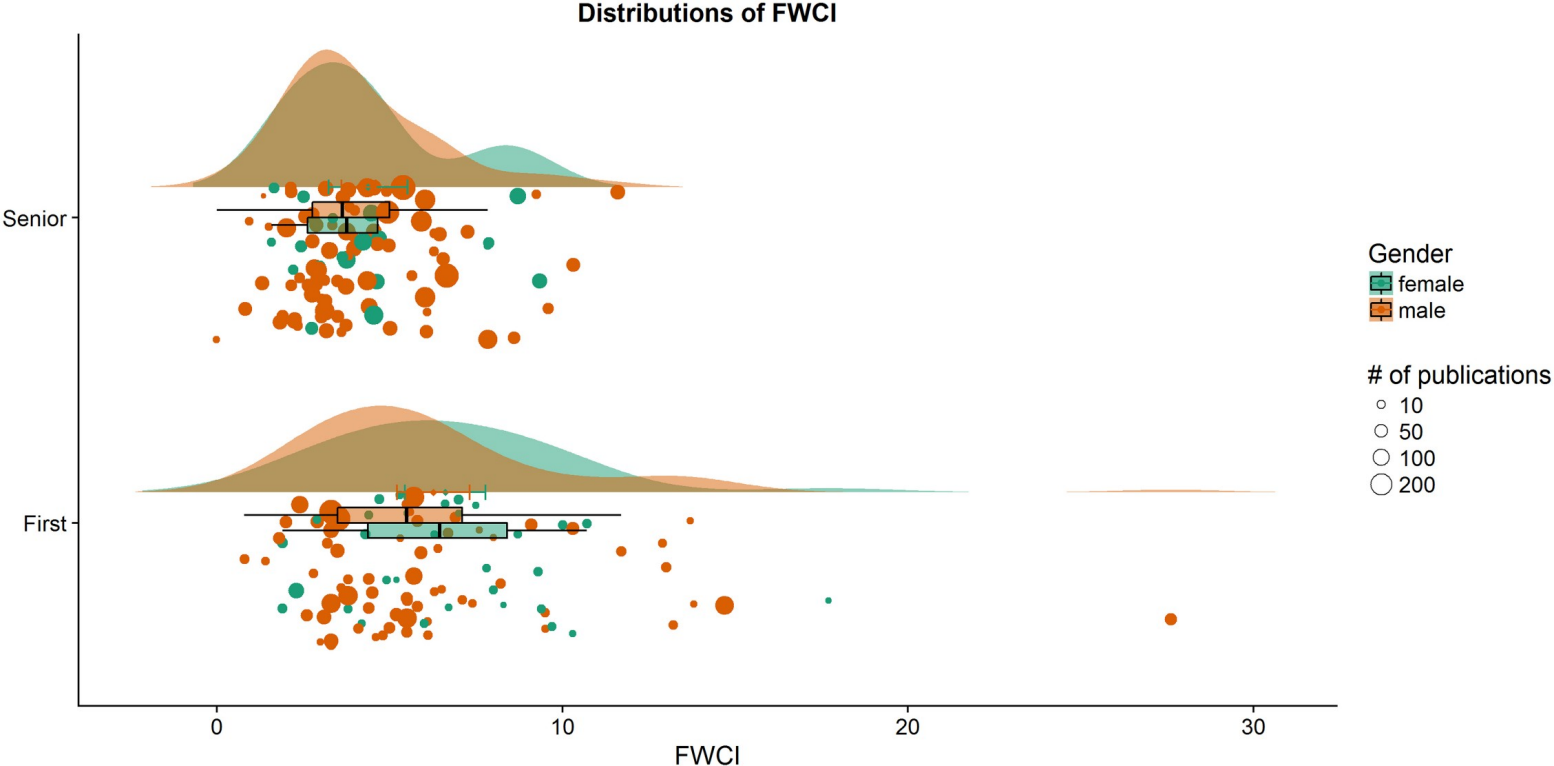
Raincloud plots of field weighted citation impact for women (green) and men (orange) first (lower plot) and senior (upper plot) authors in the top-100 database; each circle represents one author, with the size of the circle reflecting the total number of publications (see legend).

Critically, FWCI did not differ between men and women for either first or last authors (both *p*>0.49, both Cohen’s *d*<0.15, both Bayes Factor, BF_10_<0.29), indicating no significant difference in the impact of research between men and women irrespective of career stage. Fig 2 shows the gender breakdown of authors in the top-100 database for FWCI and total publications (data retrieved from SciVal). 32% of first authors and 21% of last authors were women, reflecting the underlying problem of gender disparity in science.

## Achieving high quality, gender-balanced conference programs

The data-driven approach presented here enables gender balanced speaker selection for conference programs based on scientific impact. This approach, which shows that recent high quality peer-reviewed science does not differ between men and women in the potential speaker database, should be used in conjunction with diversity and equity policy to achieve gender parity in conference programs. Importantly, this approach will improve the scientific quality at conferences by enabling the presentation of science from broader perspectives; broadening representation at scientific conferences will lead to a broadening of science, ultimately increasing the impact of science in society.

This data-driven approach to speaker selection takes a critical step in addressing the complex issue of gender disparity in STEM, and extends beyond the tools that are currently available by providing information regarding *how* to identify conference speakers. For example, online calculators can provide estimates of gender representation that is in line with base rate representation within particular disciplines, and equity and diversity policies can prescribe equitable gender representation, but neither provide any information regarding how to identify potential speakers to deliver presentations. The current approach purposefully includes established metrics of scientific impact—that are frequently used by researchers, hiring committees, and funding bodies—to improve on existing approaches to ensure high quality speaker selection. Furthermore, the combination of metrics used in the approach presented here provides a database of potential speakers with a *recent* and *relevant* high-quality publication, whilst ensuring some stability in terms of career research performance. Our approach can be used to select early- and mid-career researchers (PhD-15 year’s post-PhD) for invited presentations from the database of first authors: for example, conferences can use the database to select speakers for an ‘*Emerging researchers’* symposium. This will ensure diversity across career stage as well as gender. This is particularly important given that invited conference presentations are important for career progression, and that gender disparity in STEM is greater in senior researchers than early-career researchers [25,26]. Not only will this help career progression, but it will also broaden the scope of research presented at conferences because emerging researchers often present novel, cutting-edge data (that might not be published yet). Our approach was specifically developed for selecting invited speakers because such opportunities have a significant impact on career progression; however diversity in conference presentations selected from abstract submissions is also important, and our approach can be extended for this purpose.

Establishing the database of potential speakers using the current approach is largely automated (e.g., the exportation of publications, citations, FWCI), and the ranking of authors can be performed with simple code. For the current study, this process was neither arduous nor time-consuming. The identification of gender in the current study was somewhat time-consuming, however, this process could be automated if publishers required, and published, gender identity data: we thus urge journals to collect and make such data publicly available once articles are *in press* (not during the review process to avoid the possibility of gender information biasing the review process). (Note that this would also overcome the limitation of some journals publishing primarily author initials (e.g., *Molecular Psychiatry*), which makes it near impossible to determine gender and might disadvantage scientists who have published in those journal with the uptake of the proposed approach to speaker selection.) In the current study, the broad discipline of neuroscience was the exemplar; using keywords and the selection of specialist journals would make it possible to establish a database of potential speakers for a conference in a different discipline or for a focussed symposium within a conference. Indeed, we have previously shown that the proposed approach would be effective in the sub-discipline of brain stimulation [27]. The data from the current study are available online, and we recommend that conference program committees use these data (together with gender policy), as well as continue to collect data, to reduce gender disparity in invited speaker programs.

The current data showing that the quality of peer-reviewed science of women and men in the top 100 authors was not distinguishable, but that only 32% of first authors and 21% of last authors were women, highlights the gender bias in the publication process. Evidence suggests that women submit fewer manuscripts than men to high quality journals, have fewer manuscripts accepted for publication in high quality journals, and that publications with a senior author who is a woman are cited less than publications with a senior author who is a man [28–30]. Therefore, although the approach presented here is data-driven, the data themselves are affected by biases that negatively affect women [6,31]. Approaches to eliminate the bias in the publication process are urgently needed, and we strongly recommend that the approach presented here should be continually refined to include the most reliable and well-accepted quality metrics for STEM researchers.

It is important to note that using the western naming convention to determine gender can be insensitive to culture and changing social trends, which may lead to some errors in gender determination [32]. Although we cannot quantify the extent of such errors in the current project, it is likely the greatest impact occurs for scientists from non-western countries. In this respect, we propose it important that authors declare their gender to the journal, and that journals subsequently make these data publicly available *after* manuscript acceptance and with permission of the author. Such a collaborative initiative between journals and authors would provide the data necessary to prevent errors in gender determination and allow such statistics to be more readily available within the public domain. Related to this point, it is critical to note that achieving gender parity is not equal to achieving diversity and inclusion. The approach presented here should be extended to ensure representation of minority groups in conference programs. For example, expanding the approach to include geographical location, ethnicity, and career stage information would provide an opportunity to increase representation of minority groups in STEM.

The benefits of the current approach are twofold. First, it provides a data-driven method for selecting invited speakers (senior and early career researchers), which can have an immediate effect on reducing gender disparity at scientific conferences. Second, establishing a database of high quality researchers based on metrics of scientific impact provides convincing evidence of parity in scientific quality between men and women at the highest level. These benefits should, in turn, lead to a positive spiral in which invited speaking opportunities for women to facilitate career development through recognition of high-quality research, providing greater opportunity for collaborative outreach, which will increase the likelihood of academic promotion and leadership for women within STEM.

In light of the strengths and limitations of the current approach, we argue strongly that a combination of approaches will be most effective at reducing the persistent gender disparity. In the immediate future, we suggest that the database presented here could be used to select invited speakers for neuroscience conferences (e.g., Society for Neuroscience, Australasian Neuroscience Society, Japan Neuroscience Society, Federation of European Neurosciences). In the short-term future, we suggest that additional databases are created for use at conferences of sub-disciplines of neuroscience (e.g., International Conference of Cognitive Neuroscience) as well as other STEM disciplines. We suggest that such databases could be created by professional societies: many societies within neuroscience already have diversity and inclusion sub-committees. Given that the current approach can be largely automated and will complement and strengthen societies’ existing diversity policies, it is reasonable to assume that the motivation for uptake will be high. Following such recommendations will also increase diversity in STEM more generally, which ultimately improve scientific advancement.

## Supporting information

S1 Data Speaker database. (XLSX)

S1 Fig Histogram shows the total number of original research articles and citation for *Nature Neuroscience* from 2012–2016. Citation distributions are plotted separately for each publication year, and the dashed line represents the average number of citations for each year, which was the cut-off point used to determine authors for which gender was audited.
(PNG)

S2 Fig Histogram shows the total number of original research articles and citation for *Neuron* from 2012–2016. Citation distributions are plotted separately for each publication year, and the dashed line represents the average number of citations for each year, which was the cut-off point used to determine authors for which gender was audited.
(PNG)

S3 Fig Histogram shows the total number of original research articles and citation for *The EMBO Journal* from 2012–2016. Citation distributions are plotted separately for each publication year, and the dashed line represents the average number of citations for each year, which was the cut-off point used to determine authors for which gender was audited.
(PNG)

S4 Fig Histogram shows the total number of original research articles and citation for *Acta Neuropathologica* from 2012–2016. Citation distributions are plotted separately for each publication year, and the dashed line represents the average number of citations for each year, which was the cut-off point used to determine authors for which gender was audited.
(PNG)

S5 Fig Histogram shows the total number of original research articles and citation for *Biological Psychiatry* from 2012–2016. Citation distributions are plotted separately for each publication year, and the dashed line represents the average number of citations for each year, which was the cut-off point used to determine authors for which gender was audited.
(PNG)

S6 Fig Histogram shows the total number of original research articles and citation for *eLIFE* from 2012–2016. Citation distributions are plotted separately for each publication year, and the dashed line represents the average number of citations for each year, which was the cut-off point used to determine authors for which gender was audited.
(PNG)

S7 Fig Histogram shows the total number of original research articles and citation for *Annals of Neurology* from 2012–2016. Citation distributions are plotted separately for each publication year, and the dashed line represents the average number of citations for each year, which was the cut-off point used to determine authors for which gender was audited.
(PNG)

S8 Fig Histogram shows the total number of original research articles and citation for *PLOS Biology* from 2012–2016. Citation distributions are plotted separately for each publication year, and the dashed line represents the average number of citations for each year, which was the cut-off point used to determine authors for which gender was audited.
(PNG)

S9 Fig Histogram shows the total number of original research articles and citation for *Journal of Neuroscience* from 2012–2016. Citation distributions are plotted separately for each publication year, and the dashed line represents the average number of citations for each year, which was the cut-off point used to determine authors for which gender was audited.
(PNG)

S10 Fig Histogram shows the total number of original research articles and citation for *The Lancet Psychiatry* from 2014–2016. Citation distributions are plotted separately for each publication year, and the dashed line represents the average number of citations for each year, which was the cut-off point used to determine authors for which gender was audited.
(PNG)

S1 Table Top 100 first authors based on weighted total citation. (DOCX)

S2 Table Top 100 senior authors based on weighted total citation. (DOCX)
