# Supplementary figures and images for: Data-driven selection of conference speakers based on scientific impact to achieve gender parity
Source: PLoS One. 2019 Jul 31;14(7):e0220481. doi: 10.1371/journal.pone.0220481 (PMC6668823; doi:10.1371/journal.pone.0220481)

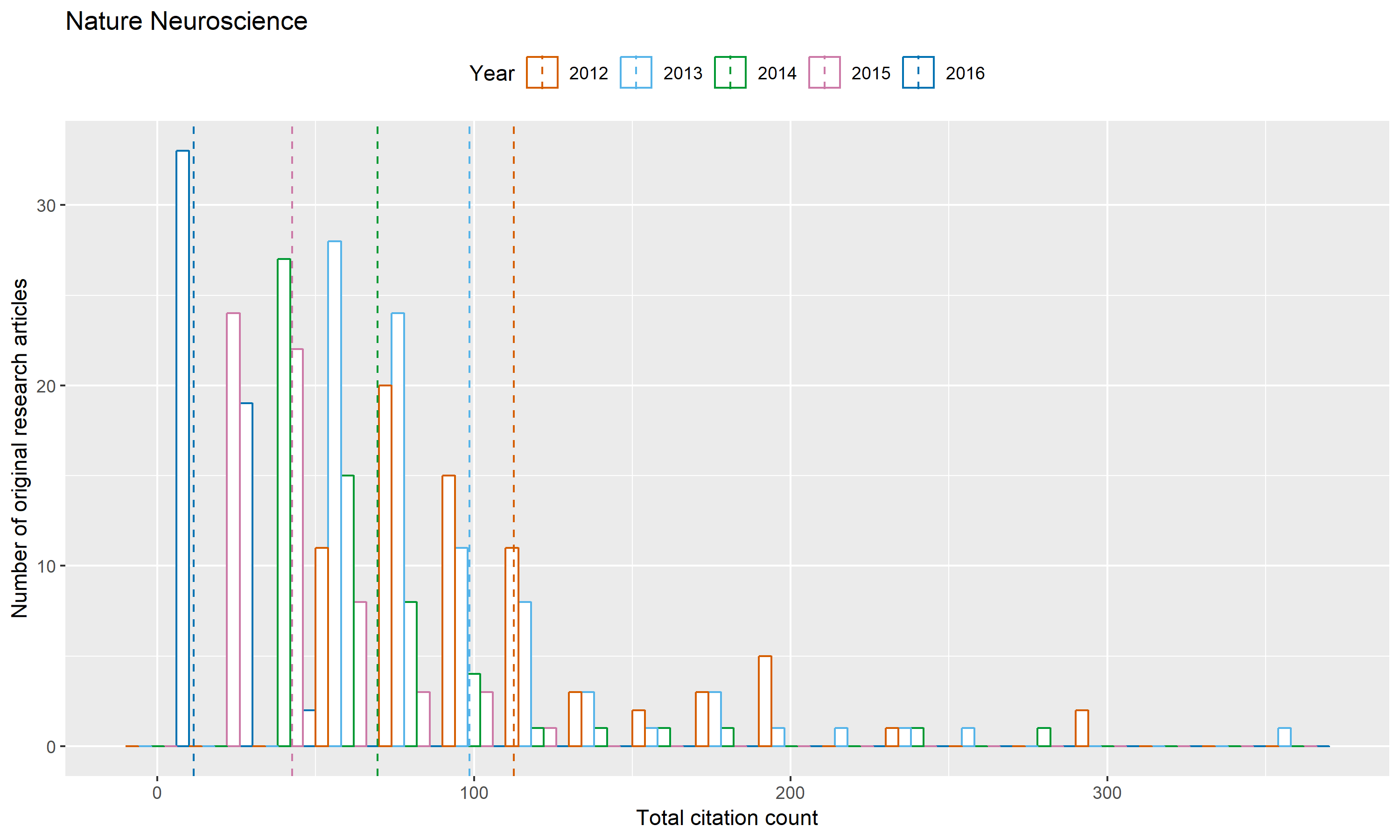

Supplement: S1 Fig — Citation distributions are plotted separately for each publication year, and the dashed line represents the average number of citations for each year, which was the cut-off point used to determine authors for which gender was audited. (PNG) [file pone.0220481.s002.png]

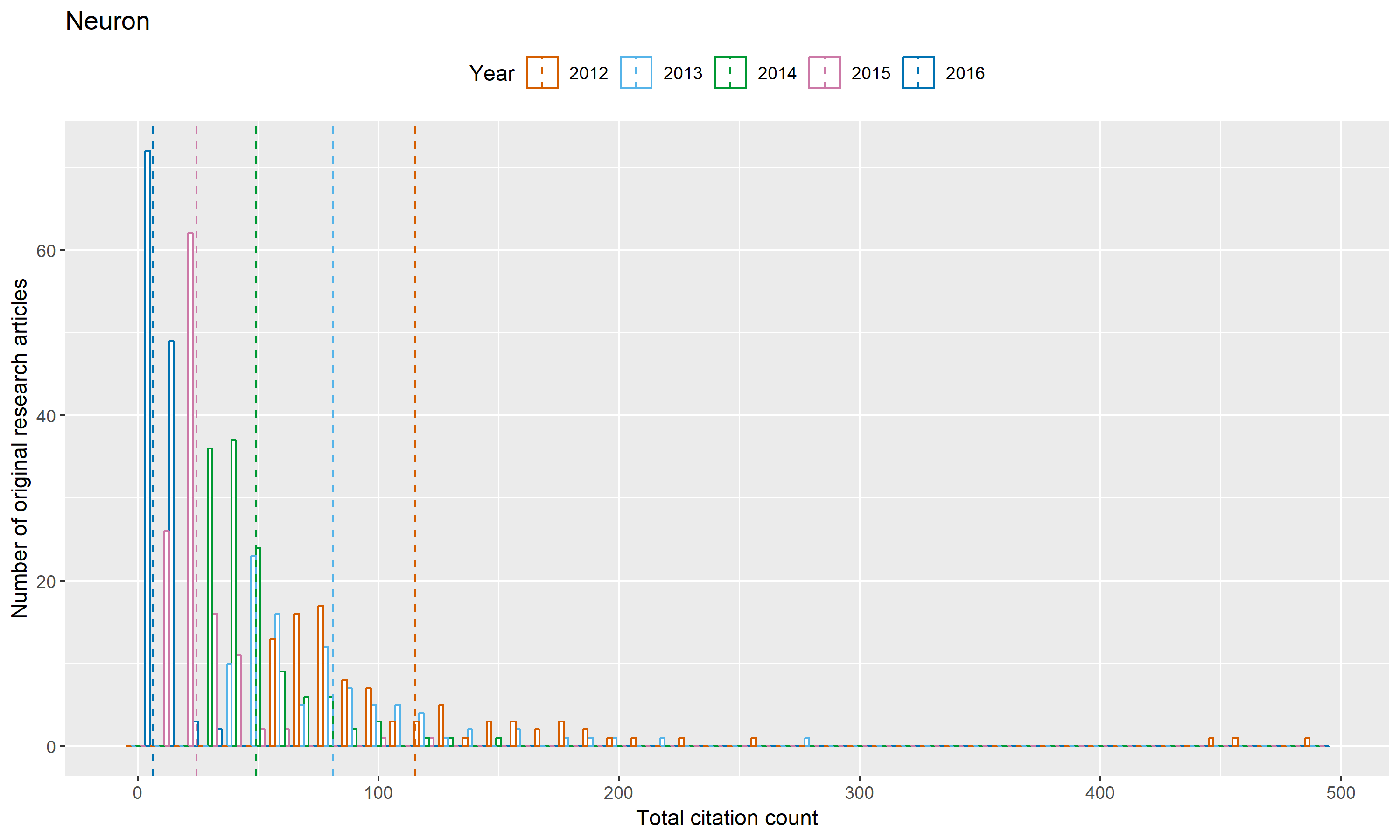

Supplement: S2 Fig — Citation distributions are plotted separately for each publication year, and the dashed line represents the average number of citations for each year, which was the cut-off point used to determine authors for which gender was audited. (PNG) [file pone.0220481.s003.png]

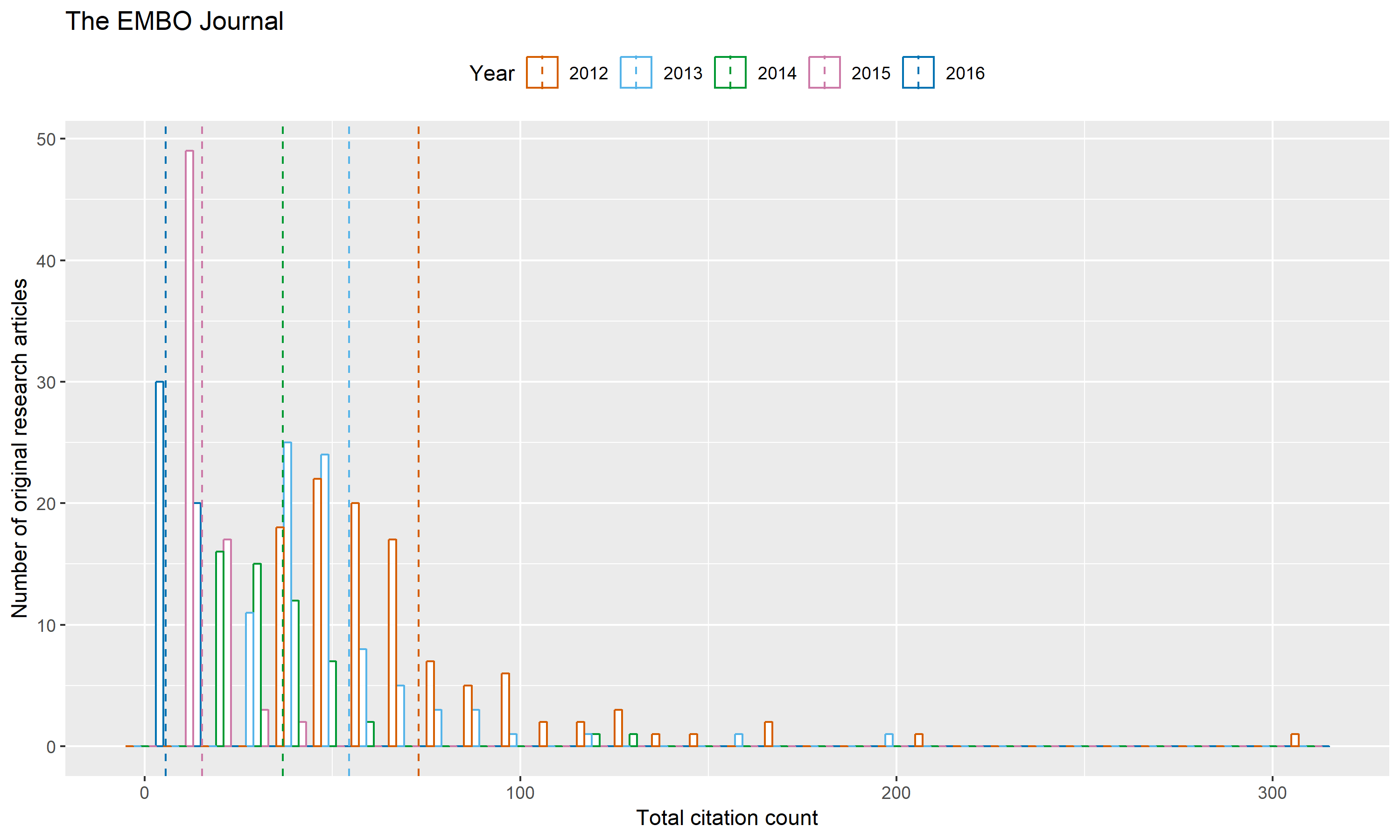

Supplement: S3 Fig — Citation distributions are plotted separately for each publication year, and the dashed line represents the average number of citations for each year, which was the cut-off point used to determine authors for which gender was audited. (PNG) [file pone.0220481.s004.png]

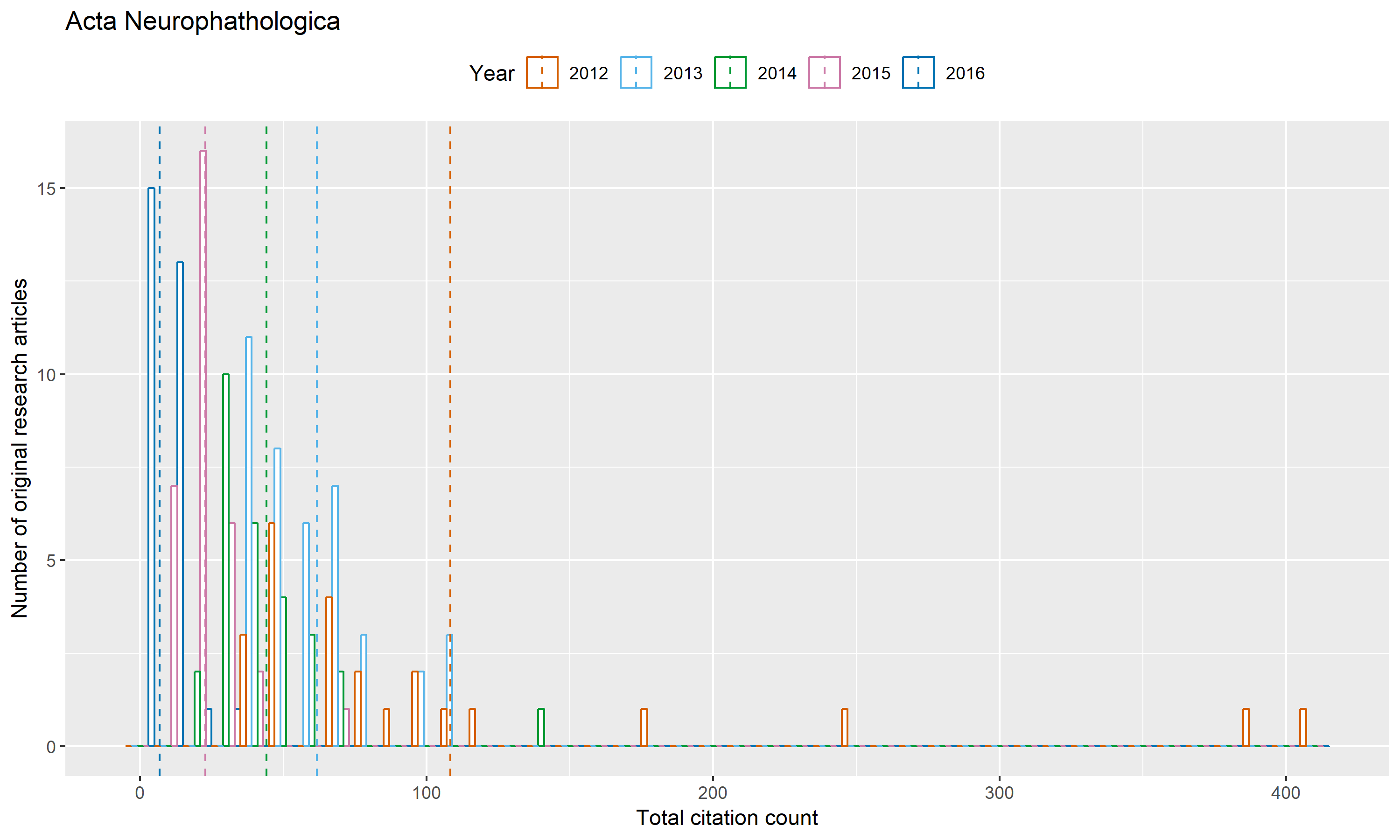

Supplement: S4 Fig — Citation distributions are plotted separately for each publication year, and the dashed line represents the average number of citations for each year, which was the cut-off point used to determine authors for which gender was audited. (PNG) [file pone.0220481.s005.png]

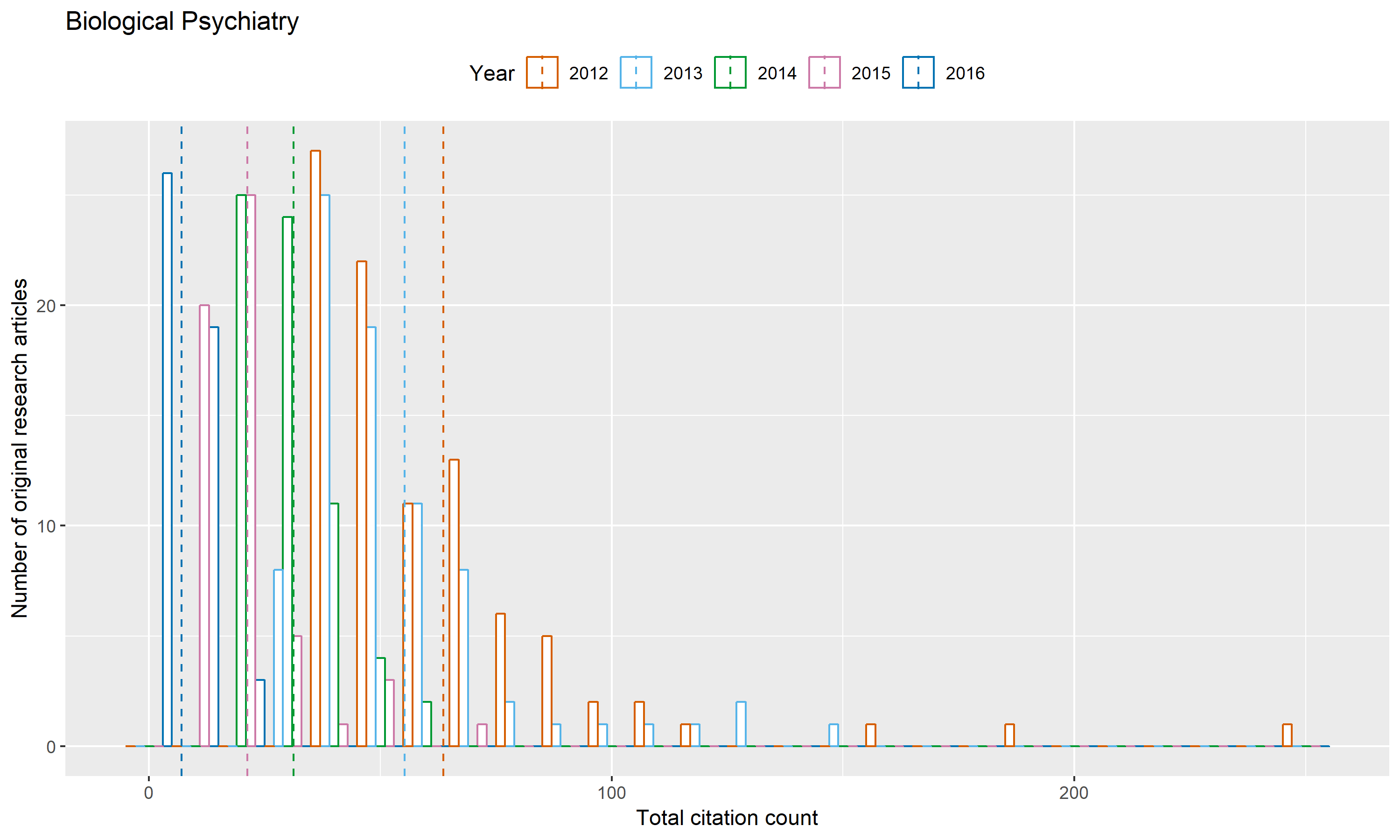

Supplement: S5 Fig — Citation distributions are plotted separately for each publication year, and the dashed line represents the average number of citations for each year, which was the cut-off point used to determine authors for which gender was audited. (PNG) [file pone.0220481.s006.png]

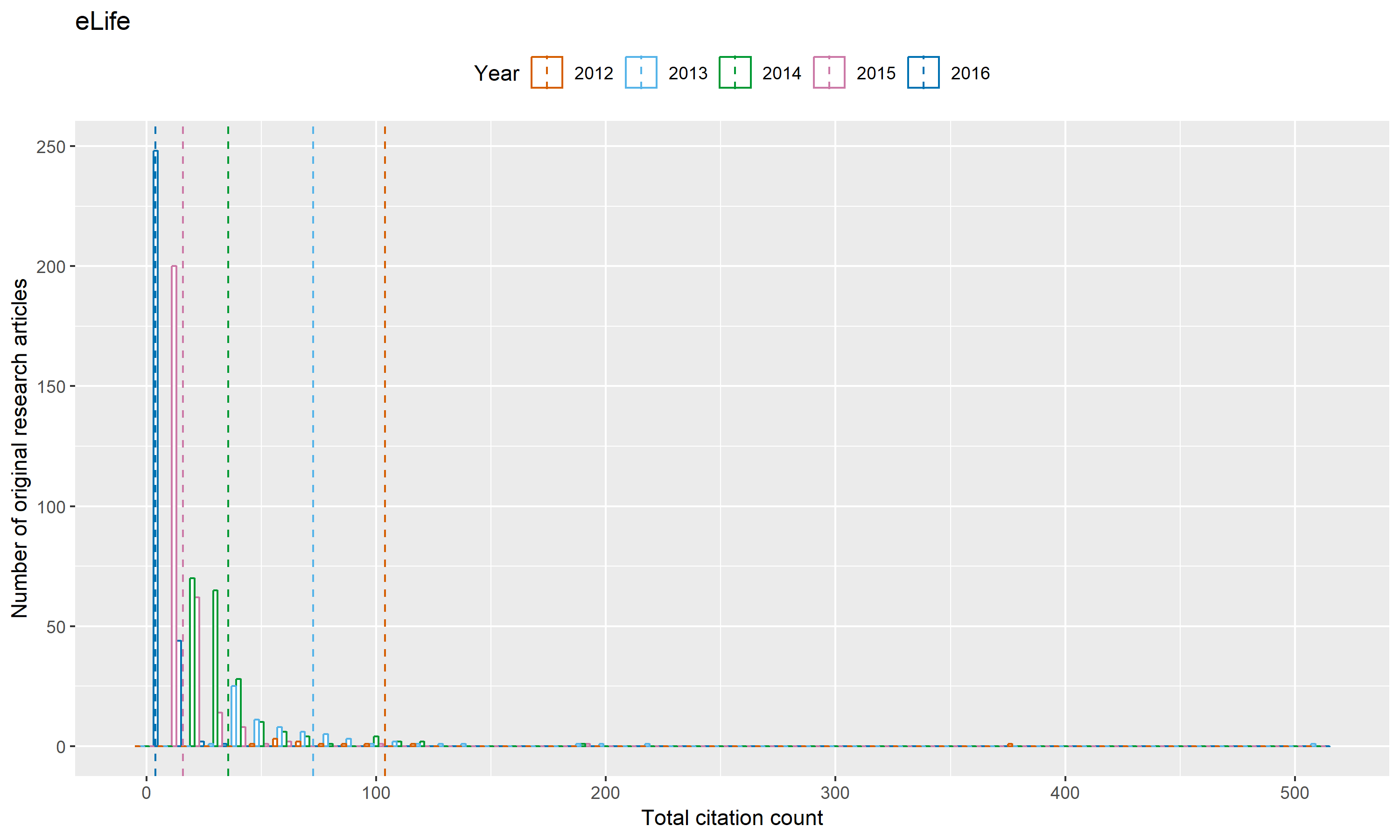

Supplement: S6 Fig — Citation distributions are plotted separately for each publication year, and the dashed line represents the average number of citations for each year, which was the cut-off point used to determine authors for which gender was audited. (PNG) [file pone.0220481.s007.png]

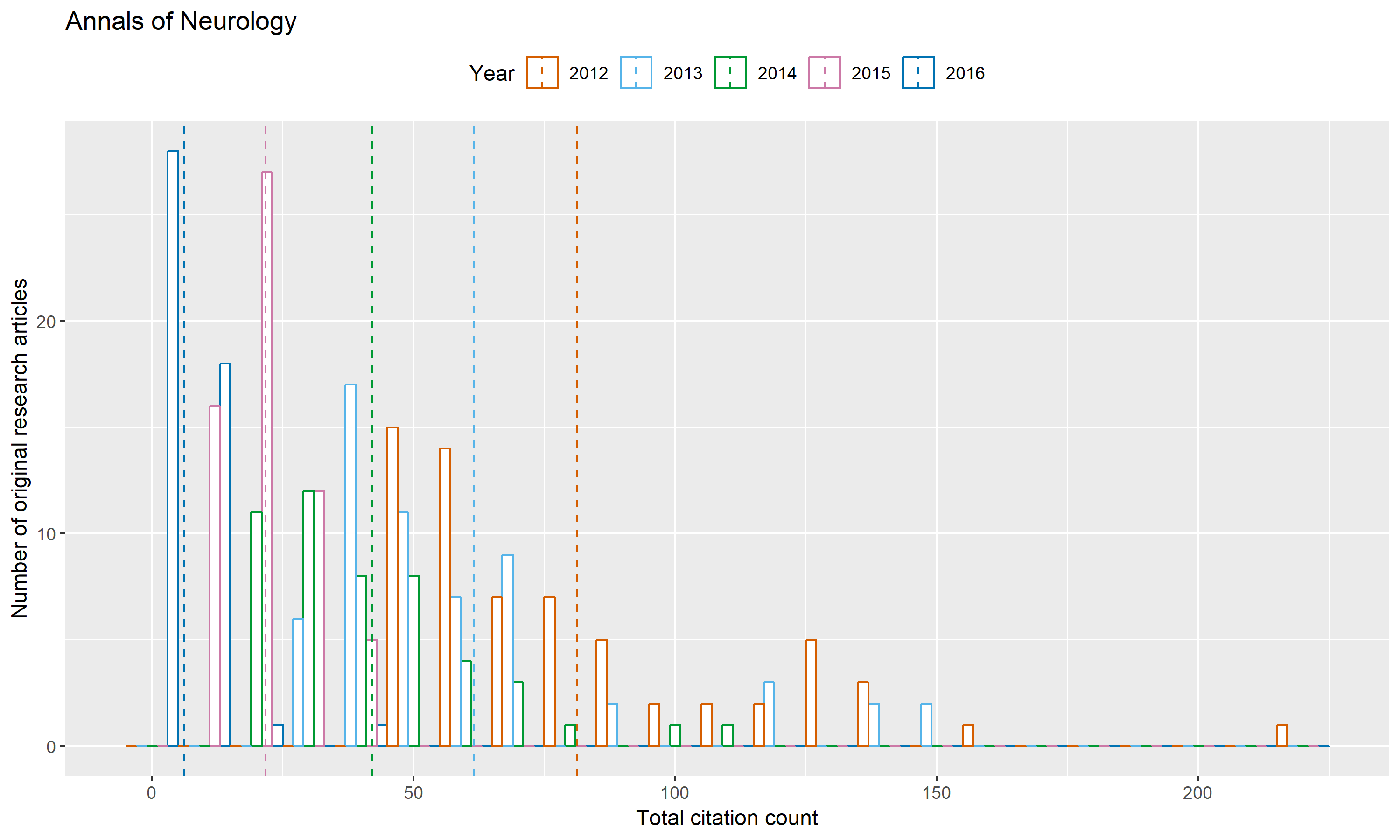

Supplement: S7 Fig — Citation distributions are plotted separately for each publication year, and the dashed line represents the average number of citations for each year, which was the cut-off point used to determine authors for which gender was audited. (PNG) [file pone.0220481.s008.png]

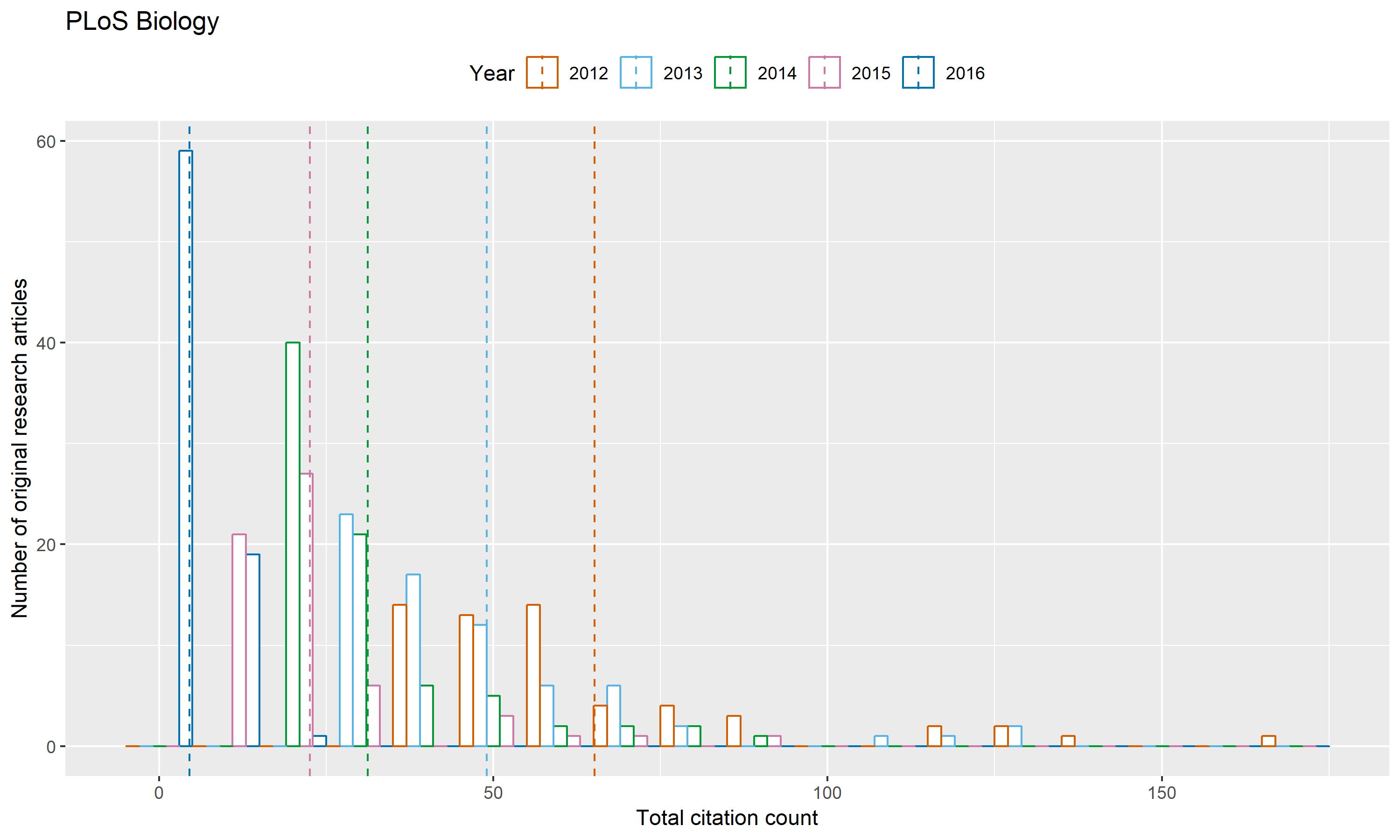

Supplement: S8 Fig — Citation distributions are plotted separately for each publication year, and the dashed line represents the average number of citations for each year, which was the cut-off point used to determine authors for which gender was audited. (PNG) [file pone.0220481.s009.png]

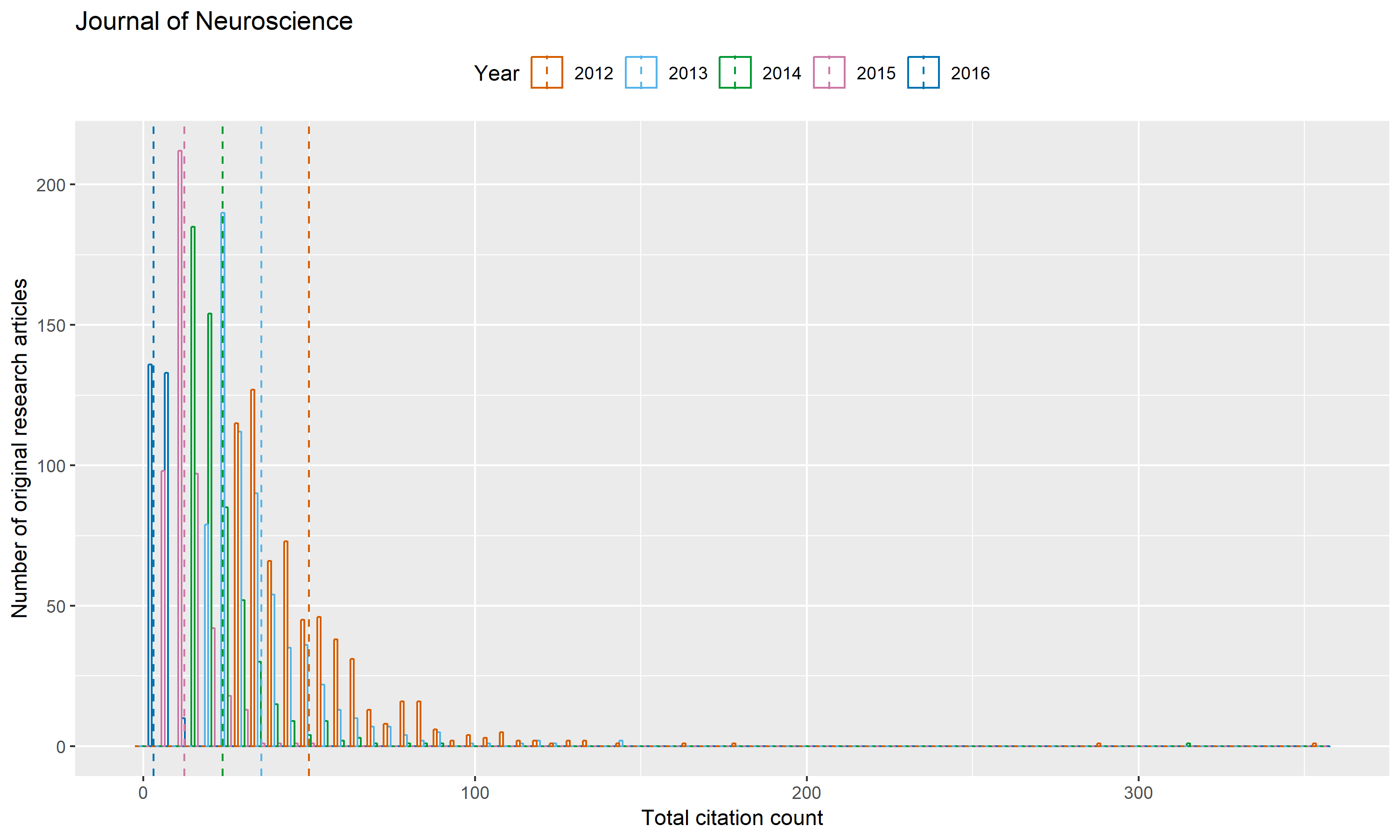

Supplement: S9 Fig — Citation distributions are plotted separately for each publication year, and the dashed line represents the average number of citations for each year, which was the cut-off point used to determine authors for which gender was audited. (PNG) [file pone.0220481.s010.png]

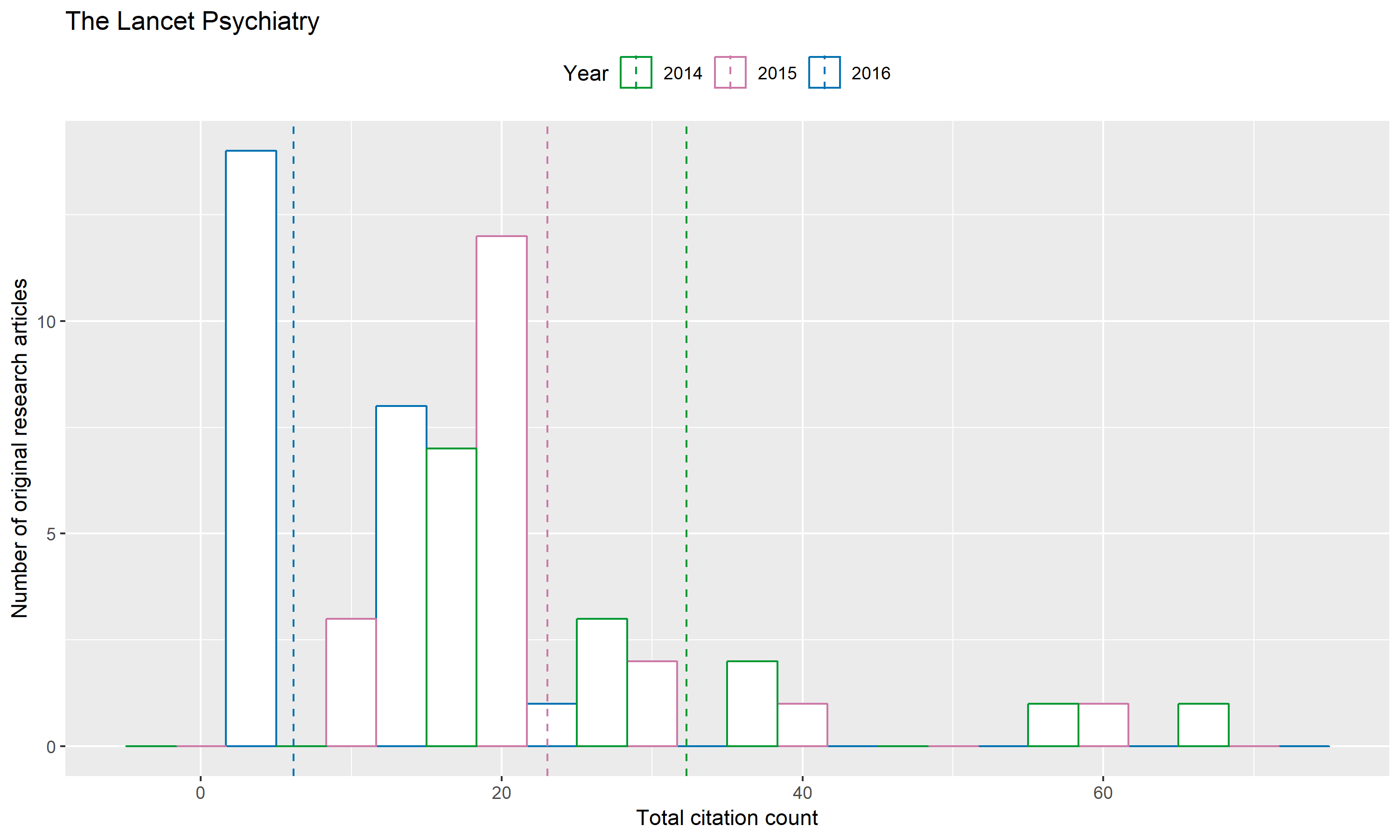

Supplement: S10 Fig — Citation distributions are plotted separately for each publication year, and the dashed line represents the average number of citations for each year, which was the cut-off point used to determine authors for which gender was audited. (PNG) [file pone.0220481.s011.png]
