# Supplementary material for: Data-driven selection of conference speakers based on scientific impact to achieve gender parity
Source: PLoS One. 2019 Jul 31;14(7):e0220481. doi: 10.1371/journal.pone.0220481 (PMC6668823; doi:10.1371/journal.pone.0220481)
Supplement: S1 Table — (DOCX) [file pone.0220481.s012.docx]

| Table 1 |  |
| --- | --- |
| Top 100 **first** authors based on weighted total citation   \| **Author** \| **Gender** \| **Journal** \| **Year** \| **wTC** \| **TC** \| **FWCI** \| **total publications** \| \| --- \| --- \| --- \| --- \| --- \| --- \| --- \| --- \| \| Iossifov, Ivan \| man \| Neuron \| 2016 \| 465.0 \| 465 \| 9.5 \| 13 \| \| Giraud, Anne-Lise \| woman \| Nature Neuroscience \| 2016 \| 305.0 \| 305 \| 1.9 \| 23 \| \| Jinek, Martin \| man \| eLife \| 2013 \| 127.3 \| 509 \| 11.7 \| 20 \| \| Schafer, Dorothy P. \| woman \| Neuron \| 2013 \| 123.3 \| 493 \| 8.0 \| 11 \| \| Akerboom, Jasper \| man \| Journal of Neuroscience \| 2014 \| 119.0 \| 357 \| 6.5 \| 8 \| \| Donnelly, Christopher J. \| man \| Neuron \| 2015 \| 109.0 \| 218 \| 7.1 \| 12 \| \| Baliki, Marwan N. \| man \| Nature Neuroscience \| 2015 \| 97.5 \| 195 \| 4.4 \| 29 \| \| Lehmann, Sabrina M. \| woman \| Nature Neuroscience \| 2015 \| 97.0 \| 194 \| 5.2 \| 2 \| \| Butovsky, Oleg \| man \| Nature Neuroscience \| 2014 \| 93.3 \| 280 \| 13.2 \| 12 \| \| Bakker, Arnold \| man \| Neuron \| 2015 \| 91.0 \| 182 \| 3.1 \| 69 \| \| Klengel, Torsten \| man \| Nature Neuroscience \| 2013 \| 90.5 \| 362 \| 5.8 \| 36 \| \| Hickman, Suzanne E. \| woman \| Nature Neuroscience \| 2015 \| 89.0 \| 178 \| 6.3 \| 6 \| \| Watabe-Uchida, Mitsuko \| woman \| Neuron \| 2014 \| 88.0 \| 264 \| 6.6 \| 8 \| \| Kierdorf, Katrin \| woman \| Nature Neuroscience \| 2014 \| 81.7 \| 245 \| 9.3 \| 14 \| \| Montine, Thomas J. \| man \| Acta Neuropathologica \| 2012 \| 81.6 \| 408 \| 14.7 \| 131 \| \| Zhang, Ye; Chen \| unkown \| Journal of Neuroscience \| 2013 \| 78.8 \| 315 \| 0.1 \| 5 \| \| Taylor, Michael D. \| man \| Acta Neuropathologica \| 2012 \| 77.8 \| 389 \| 3.3 \| 247 \| \| Madisen, Linda \| woman \| Nature Neuroscience \| 2013 \| 74.3 \| 297 \| 9.4 \| 10 \| \| Crary, John F. \| man \| Acta Neuropathologica \| 2015 \| 70.0 \| 140 \| 8.2 \| 18 \| \| Hipp, Joerg F. \| man \| Nature Neuroscience \| 2014 \| 64.7 \| 194 \| 2.8 \| 14 \| \| Dulvy, Nicholas K. \| man \| eLife \| 2014 \| 64.3 \| 193 \| 5.9 \| 42 \| \| Usoskin, Dmitry \| man \| Nature Neuroscience \| 2015 \| 63.0 \| 126 \| 13.7 \| 29 \| \| Bazzini, Ariel A. \| man \| EMBO Journal \| 2015 \| 63.0 \| 126 \| 6.1 \| 8 \| \| Settembre, Carmine \| man \| EMBO Journal \| 2012 \| 63.0 \| 315 \| 4.4 \| 3 \| \| Griciuc, Ana \| woman \| Neuron \| 2014 \| 62.3 \| 187 \| 7.5 \| 48 \| \| Dannlowski, Udo \| man \| Biological Psychiatry \| 2013 \| 62.3 \| 249 \| 2.9 \| 3 \| \| Kool, Marcel \| man \| Acta Neuropathologica \| 2013 \| 62.0 \| 248 \| 5.7 \| 112 \| \| Dias, Brian G. \| man \| Nature Neuroscience \| 2013 \| 61.0 \| 244 \| 4.8 \| 15 \| \| Montagne, Axel \| man \| Neuron \| 2015 \| 60.0 \| 120 \| 3.3 \| 20 \| \| Kravitz, Alexxai V. \| man \| Nature Neuroscience \| 2013 \| 59.8 \| 239 \| 3.2 \| 21 \| \| Zamanian, Jennifer L. \| woman \| Journal of Neuroscience \| 2012 \| 58.0 \| 290 \| 17.7 \| 2 \| \| van Zessen, Ruud \| man \| Neuron \| 2014 \| 56.7 \| 170 \| 5.3 \| 5 \| \| Ash, Peter E. A. \| man \| Neuron \| 2012 \| 56.2 \| 281 \| 6.3 \| 10 \| \| Leech, Robert \| man \| Journal of Neuroscience \| 2014 \| 55.0 \| 165 \| 3.3 \| 68 \| \| Scher, Jose U. \| man \| eLife \| 2013 \| 54.0 \| 216 \| 6.7 \| 24 \| \| Miron, Veronique E. \| woman \| Nature Neuroscience \| 2012 \| 53.8 \| 269 \| 5.3 \| 8 \| \| MacLeod, David A. \| man \| Neuron \| 2014 \| 53.7 \| 161 \| 1.4 \| 8 \| \| Yu, Timothy W. \| man \| Neuron \| 2014 \| 53.0 \| 159 \| 6.9 \| 4 \| \| Cajigas, Ivan J. \| man \| Neuron \| 2014 \| 53.0 \| 159 \| 3.0 \| 27 \| \| Grienberger, Christine \| woman \| Neuron \| 2013 \| 52.3 \| 209 \| 2.9 \| 9 \| \| Plavina, Tatiana \| woman \| Annals of Neurology \| 2015 \| 52.0 \| 104 \| 10.7 \| 13 \| \| Bastos, Andre Moraes \| man \| Neuron \| 2015 \| 52.0 \| 104 \| 6.1 \| 14 \| \| Lagier-Tourenne, Clotilde \| woman \| Nature Neuroscience \| 2013 \| 51.0 \| 204 \| 7.0 \| 17 \| \| Sauvageau, Martin \| man \| eLife \| 2013 \| 49.8 \| 199 \| 12.9 \| 11 \| \| Iba, Michiyo \| unkown \| Journal of Neuroscience \| 2014 \| 49.0 \| 147 \| 6.5 \| 9 \| \| Kraemer, Moritz U. G. \| man \| eLife \| 2015 \| 48.5 \| 97 \| 13.0 \| 19 \| \| Brettschneider, Johannes \| man \| Annals of Neurology \| 2014 \| 48.0 \| 144 \| 5.5 \| 33 \| \| Ke, Meng-Tsen \| woman \| Nature Neuroscience \| 2013 \| 47.3 \| 189 \| 5.5 \| 3 \| \| Gapp, Katharina \| woman \| Nature Neuroscience \| 2013 \| 47.0 \| 188 \| 6.0 \| 10 \| \| Knobloch, H. Sophie \| woman \| Neuron \| 2012 \| 46.8 \| 234 \| 7.0 \| 15 \| \| Agarwal, Vikram \| man \| eLife \| 2013 \| 46.8 \| 187 \| 0.8 \| 3 \| \| Freischmidt, Axel \| man \| Nature Neuroscience \| 2015 \| 45.5 \| 91 \| 3.6 \| 14 \| \| Kordasiewicz, Holly B. \| woman \| Neuron \| 2013 \| 44.8 \| 179 \| 4.9 \| 9 \| \| Balbas, Minna D. \| woman \| eLife \| 2014 \| 44.7 \| 134 \| 10.3 \| 3 \| \| Jack, Clifford R., Jr. \| man \| Annals of Neurology \| 2012 \| 44.6 \| 223 \| 3.5 \| 289 \| \| Young, Kaylene M. \| woman \| Neuron \| 2013 \| 44.0 \| 176 \| 1.9 \| 17 \| \| Atallah, Bassam V. \| man \| Neuron \| 2013 \| 43.5 \| 174 \| 4.6 \| 6 \| \| Garg, Abhishek D. \| man \| EMBO Journal \| 2013 \| 43.0 \| 172 \| 5.2 \| 50 \| \| Cole, Michael W. \| man \| Nature Neuroscience \| 2012 \| 41.0 \| 205 \| 5.5 \| 18 \| \| Steentoft, Catharina \| woman \| EMBO Journal \| 2012 \| 41.0 \| 205 \| 4.3 \| 28 \| \| Pigott, David M. \| man \| eLife \| 2014 \| 40.0 \| 120 \| 27.6 \| 17 \| \| Bosman, Conrado A. \| man \| Neuron \| 2012 \| 40.0 \| 200 \| 3.8 \| 33 \| \| Zarate, Carlos A., Jr. \| man \| Biological Psychiatry \| 2012 \| 39.0 \| 195 \| 3.3 \| 145 \| \| Threlfell, Sarah \| woman \| Neuron \| 2012 \| 37.4 \| 187 \| 3.3 \| 9 \| \| Mendell, Jerry R. \| man \| Annals of Neurology \| 2013 \| 37.3 \| 149 \| 3.5 \| 62 \| \| Orenstein, Samantha J. \| woman \| Nature Neuroscience \| 2013 \| 37.0 \| 148 \| 8.3 \| 2 \| \| Wheaton, William W. \| man \| eLife \| 2014 \| 36.0 \| 108 \| 13.8 \| 10 \| \| Guo, Junjie U. \| man \| Nature Neuroscience \| 2013 \| 36.0 \| 144 \| 6.4 \| 4 \| \| Dong-Anh Khuong-Quang \| woman \| Acta Neuropathologica \| 2012 \| 35.6 \| 178 \| 10.0 \| 12 \| \| Zaki, Jamil \| man \| Nature Neuroscience \| 2012 \| 35.0 \| 175 \| 1.8 \| 34 \| \| Noble, Kimberly G. \| woman \| Nature Neuroscience \| 2015 \| 34.5 \| 69 \| 4.7 \| 17 \| \| Kijas, James W. \| man \| PLoS Biology \| 2012 \| 34.0 \| 170 \| 2.6 \| 36 \| \| Tan, Kelly R. \| woman \| Neuron \| 2012 \| 32.4 \| 162 \| 4.2 \| 7 \| \| Faber, Catharina G. \| woman \| Annals of Neurology \| 2012 \| 32.2 \| 161 \| 2.3 \| 82 \| \| Deplus, Rachel \| woman \| EMBO Journal \| 2012 \| 32.0 \| 160 \| 3.8 \| 81 \| \| Mouillot, David \| man \| PLoS Biology \| 2013 \| 32.0 \| 128 \| 3.3 \| 10 \| \| Hughes, Ethan G. \| man \| Nature Neuroscience \| 2013 \| 31.5 \| 126 \| 7.6 \| 4 \| \| McLelland, Gian-Luca \| man \| EMBO Journal \| 2013 \| 30.3 \| 121 \| 9.5 \| 7 \| \| Kessler, Ronald C. \| man \| Biological Psychiatry \| 2012 \| 30.2 \| 151 \| 3.8 \| 152 \| \| Rodgers, Ali B. \| unkown \| Journal of Neuroscience \| 2012 \| 29.4 \| 147 \| 7.3 \| 11 \| \| Ziv, Yaniv \| man \| Nature Neuroscience \| 2012 \| 29.4 \| 147 \| 4.5 \| 40 \| \| Elmore, Monica R. P. \| woman \| Neuron \| 2012 \| 29.4 \| 147 \| 4.4 \| 5 \| \| De Jager, Philip L. \| man \| Nature Neuroscience \| 2013 \| 29.0 \| 116 \| 5.5 \| 152 \| \| Recasens, Ariadna \| woman \| Annals of Neurology \| 2013 \| 28.8 \| 115 \| 6.7 \| 5 \| \| Musiek, Erik S. \| man \| Nature Neuroscience \| 2014 \| 27.3 \| 82 \| 5.8 \| 25 \| \| Erny, Daniel \| man \| Nature Neuroscience \| 2013 \| 27.0 \| 108 \| 7.4 \| 8 \| \| Paz, Jeanne T. \| woman \| Nature Neuroscience \| 2012 \| 25.4 \| 127 \| 7.8 \| 8 \| \| Cole, Michael W. \| man \| Neuron \| 2012 \| 24.6 \| 123 \| 5.5 \| 28 \| \| O'Dushlaine, Colm \| man \| Nature Neuroscience \| 2012 \| 20.4 \| 102 \| 10.3 \| 10 \| \| Sojka, Dorothy K. \| woman \| eLife \| 2012 \| 20.4 \| 102 \| 9.7 \| 43 \| \| Scheres, Sjors H. W. \| man \| eLife \| 2012 \| 20.2 \| 101 \| 9.1 \| 39 \| \| Alami, Nael H. \| man \| Neuron \| 2012 \| 19.6 \| 98 \| 8.0 \| 3 \| \| Deisseroth, Karl \| man \| Nature Neuroscience \| 2013 \| 19.3 \| 77 \| 5.7 \| 186 \| \| Cannon, Tyrone D. \| man \| Biological Psychiatry \| 2013 \| 16.5 \| 66 \| 2.4 \| 112 \| \| Sorge, Robert E. \| man \| Nature Neuroscience \| 2012 \| 16.2 \| 81 \| 4.1 \| 20 \| \| Herrup, Karl \| man \| Nature Neuroscience \| 2012 \| 14.0 \| 70 \| 2.0 \| 38 \| \| Reuss, David E. \| man \| Acta Neuropathologica \| 2012 \| 13.8 \| 69 \| 5.0 \| 29 \| \| Khodagholy, Dion \| man \| Nature Neuroscience \| 2012 \| 13.0 \| 65 \| 5.5 \| 18 \| \| Tasic, Bosiljka \| woman \| Nature Neuroscience \| 2013 \| 10.0 \| 40 \| 8.7 \| 9 \| \| Hamid, Arif A. \| man \| Nature Neuroscience \| 2012 \| 6.6 \| 33 \| 5.6 \| 6 \| | |

*FWCI: field-weighted citation impact; TC: total citation; wTC; weighted total citation*
