# Supplementary material for: Data-driven selection of conference speakers based on scientific impact to achieve gender parity
Source: PLoS One. 2019 Jul 31;14(7):e0220481. doi: 10.1371/journal.pone.0220481 (PMC6668823; doi:10.1371/journal.pone.0220481)
Supplement: S2 Table — (DOCX) [file pone.0220481.s013.docx]

| Table 2 | | |  |  |  |  |  |  |  |
| --- | --- | --- | --- | --- | --- | --- | --- | --- | --- |
| Top 100 **senior** authors based on weighted total citation | | | | | | | | |  |
| **Author** | **Gender** | **Journal** | | **Year** | **wTC** | **TC** | **FWCI** | **total publications** | |
| Doudna, Jennifer | woman | eLife | | 2013 | 127.3 | 509 | 8.7 | 93 | |
| Wu, Jia Qian | woman | Journal of Neuroscience | | 2014 | 105.0 | 315 | 7.8 | 7 | |
| Stevens, Beth | woman | Neuron | | 2012 | 98.6 | 493 | 7.9 | 28 | |
| Bartel, David P. | man | eLife | | 2015 | 93.5 | 187 | 9.6 | 26 | |
| Weiner, Howard L. | man | Nature Neuroscience | | 2014 | 93.3 | 280 | 3.8 | 119 | |
| Wigler, Michael | man | Neuron | | 2012 | 93.0 | 465 | 4.9 | 25 | |
| Binder, Elisabeth B. | woman | Nature Neuroscience | | 2013 | 90.5 | 362 | 4.2 | 118 | |
| Hyman, Bradley T. | man | Neuron | | 2012 | 89.2 | 446 | 4.4 | 139 | |
| Hyman, Bradley T. | man | Acta Neuropathologica | | 2012 | 81.6 | 408 | 4.4 | 139 | |
| Ressier, Kerry J. | man | Nature Neuroscience | | 2014 | 81.3 | 244 | 3.1 | 151 | |
| Pfister, Stefan M. | man | Acta Neuropathologica | | 2012 | 77.8 | 389 | 6.0 | 174 | |
| Li, Wenhui | man | eLife | | 2012 | 76.0 | 380 | 0.8 | 57 | |
| Looger, Loren L. | man | Journal of Neuroscience | | 2012 | 71.4 | 357 | 5.0 | 54 | |
| Petrucelli, Leonard | man | Neuron | | 2013 | 70.3 | 281 | 6.6 | 49 | |
| Ffrench-Constant, Charles | man | Nature Neuroscience | | 2013 | 67.3 | 269 | 3.0 | 24 | |
| White, William T. | man | eLife | | 2014 | 64.3 | 193 | 2.3 | 62 | |
| Ballabio, Andrea | man | EMBO Journal | | 2012 | 63.0 | 315 | 10.3 | 56 | |
| Mansuy, Isabelle M. | woman | Nature Neuroscience | | 2014 | 62.7 | 188 | 2.5 | 40 | |
| Prinz, Marco | man | Nature Neuroscience | | 2013 | 61.3 | 245 | 2.9 | 110 | |
| Barres, Ben A. | man | Journal of Neuroscience | | 2012 | 58.0 | 290 | 6.5 | 61 | |
| Scanziani, Massimo | man | Nature Neuroscience | | 2013 | 56.8 | 227 | 6.3 | 16 | |
| Rothstein, Jeffrey D. | man | Neuron | | 2013 | 54.5 | 218 | 4.7 | 60 | |
| Littman, Dan R. | man | eLife | | 2013 | 54.0 | 216 | 6.1 | 48 | |
| Uchida, Naoshige | man | Neuron | | 2012 | 52.8 | 264 | 3.9 | 24 | |
| Gross, Cornelius T. | man | Nature Neuroscience | | 2014 | 51.7 | 155 | 2.2 | 30 | |
| Braver, Todd S. | man | Nature Neuroscience | | 2013 | 51.3 | 205 | 3.5 | 43 | |
| Clausen, Henrik | man | EMBO Journal | | 2013 | 51.3 | 205 | 2.6 | 67 | |
| Kugel, Harald | man | Biological Psychiatry | | 2012 | 49.8 | 249 | 2.3 | 75 | |
| Rinn, John L. | man | eLife | | 2013 | 49.8 | 199 | 11.6 | 67 | |
| Pfister, Stefan M. | man | Acta Neuropathologica | | 2012 | 49.6 | 248 | 6.0 | 174 | |
| Higuchi, Makoto | unknown | Neuron | | 2013 | 49.5 | 198 | 1.7 | 62 | |
| Green, Kim N. | man | Neuron | | 2014 | 49.0 | 147 | 5.7 | 23 | |
| Scheres, Sjors H. W. | man | eLife | | 2013 | 48.3 | 193 | 8.6 | 39 | |
| Song, Hongjun | man | Nature Neuroscience | | 2014 | 48.0 | 144 | 0.0 | 4 | |
| Imai, Takeshi | man | Nature Neuroscience | | 2013 | 47.3 | 189 | 0.9 | 8 | |
| Grinevich, Valery | woman | Neuron | | 2012 | 46.8 | 234 | 3.4 | 19 | |
| Tanzi, Rudolph E. | man | Neuron | | 2013 | 46.8 | 187 | 3.8 | 76 | |
| Nelson, Peter T. | man | Acta Neuropathologica | | 2014 | 46.7 | 140 | 5.0 | 67 | |
| Petersen, Ronald C. | man | Annals of Neurology | | 2012 | 44.6 | 223 | 5.4 | 283 | |
| El Khoury, Joseph | man | Nature Neuroscience | | 2013 | 44.5 | 178 | 3.6 | 15 | |
| Janak, Patricia H. | woman | Nature Neuroscience | | 2013 | 44.5 | 178 | 3.6 | 30 | |
| Richardson, William D. | man | Neuron | | 2013 | 44.0 | 176 | 3.9 | 24 | |
| Konnerth, Arthur | man | Neuron | | 2012 | 41.8 | 209 | 2.1 | 30 | |
| Clevers, Hans | man | EMBO Journal | | 2012 | 41.2 | 206 | 5.9 | 183 | |
| Abeliovich, Asa | man | Neuron | | 2013 | 40.3 | 161 | 3.8 | 17 | |
| Fries, Pascal | man | Neuron | | 2012 | 40.0 | 200 | 3.5 | 40 | |
| Fuks, Francois | man | EMBO Journal | | 2013 | 40.0 | 160 | 3.1 | 27 | |
| Tsien, Roger Y. | man | Nature Neuroscience | | 2013 | 40.0 | 160 | 2.9 | 59 | |
| Walsh, Christopher A. | man | Neuron | | 2013 | 39.8 | 159 | 4.5 | 76 | |
| Lindemann, Lothar | unknown | Neuron | | 2012 | 39.0 | 195 | 3.0 | 16 | |
| Luckenbaugh, David A. | man | Biological Psychiatry | | 2012 | 39.0 | 195 | 2.8 | 59 | |
| Brown, Peter | man | Annals of Neurology | | 2013 | 38.5 | 154 | 2.8 | 90 | |
| Xavier, Miguel | man | Biological Psychiatry | | 2013 | 37.8 | 151 | 1.9 | 36 | |
| Cragg, Stephanie J. | woman | Neuron | | 2012 | 37.4 | 187 | 2.2 | 18 | |
| Kaye, Edward M. | man | Annals of Neurology | | 2013 | 37.3 | 149 | 6.1 | 9 | |
| Cuervo, Ana Maria | woman | Nature Neuroscience | | 2013 | 37.0 | 148 | 9.3 | 73 | |
| Bale, Tracy L. | woman | Journal of Neuroscience | | 2013 | 36.8 | 147 | 3.0 | 33 | |
| Lee, Virginia M-Y | woman | Journal of Neuroscience | | 2013 | 36.8 | 147 | 4.5 | 141 | |
| Schnitzer, Mark J. | man | Nature Neuroscience | | 2013 | 36.8 | 147 | 3.7 | 43 | |
| Seeley, William W. | man | Neuron | | 2012 | 36.6 | 183 | 1.5 | 6 | |
| Gallagher, Michela | woman | Neuron | | 2012 | 36.4 | 182 | 1.7 | 24 | |
| Trojanowski, John Q. | man | Annals of Neurology | | 2013 | 36.0 | 144 | 6.7 | 261 | |
| Cleveland, Don W. | man | Neuron | | 2012 | 35.8 | 179 | 3.7 | 65 | |
| Buzsáki, György | man | Journal of Neuroscience | | 2012 | 35.6 | 178 | 4.0 | 76 | |
| Hawkins, Cynthia | woman | Acta Neuropathologica | | 2012 | 35.6 | 178 | 3.8 | 115 | |
| Birbaumer, Niels | man | Annals of Neurology | | 2013 | 35.5 | 142 | 2.0 | 140 | |
| Vanderhaeghen, Pierre | man | Neuron | | 2013 | 35.0 | 140 | 2.4 | 24 | |
| Scanziani, Massimo | man | Neuron | | 2012 | 34.8 | 174 | 6.3 | 16 | |
| Agostinis, Patrizia | woman | EMBO Journal | | 2012 | 34.4 | 172 | 4.7 | 82 | |
| Dalrymple, Brian | man | PLoS Biology | | 2012 | 34.0 | 170 | 2.3 | 20 | |
| Nowak, Martin A. | man | eLife | | 2013 | 34.0 | 136 | 2.8 | 113 | |
| Stuber, Garret D. | man | Neuron | | 2012 | 34.0 | 170 | 3.2 | 49 | |
| Tsai, Li-Huei | woman | Neuron | | 2013 | 34.0 | 136 | 4.6 | 77 | |
| Rudy, Bernardo | man | Nature Neuroscience | | 2013 | 33.5 | 134 | 4.0 | 23 | |
| Sawyers, Charles L. | man | eLife | | 2013 | 33.5 | 134 | 7.3 | 52 | |
| De Strooper, Bart | man | EMBO Journal | | 2012 | 33.4 | 167 | 3.7 | 96 | |
| Sharp, David J. | man | Journal of Neuroscience | | 2012 | 33.0 | 165 | 1.4 | 1 | |
| Strittmatter, Stephen M. | man | Neuron | | 2013 | 33.0 | 132 | 2.7 | 50 | |
| Lüscher, Christian H.R. | man | Neuron | | 2012 | 32.4 | 162 | 3.0 | 42 | |
| Merkies, Ingemar S. J. | unknown | Annals of Neurology | | 2012 | 32.2 | 161 | 2.6 | 78 | |
| Schuman, Erin M. | woman | Neuron | | 2012 | 31.8 | 159 | 2.4 | 34 | |
| Weiner, Michael | man | Neuron | | 2012 | 31.6 | 158 | 5.0 | 241 | |
| Yao, Shuqiao | man | Biological Psychiatry | | 2012 | 31.2 | 156 | 1.3 | 62 | |
| Stanton, Lawrence W. | man | EMBO Journal | | 2012 | 31.0 | 155 | 2.2 | 38 | |
| Gallant, Jack L. | man | Neuron | | 2012 | 30.0 | 150 | 2.9 | 16 | |
| Ances, Beau M. | man | Journal of Neuroscience | | 2012 | 29.2 | 146 | 3.2 | 74 | |
| Bonci, Antonello | man | Neuron | | 2012 | 29.2 | 146 | 2.8 | 61 | |
| Walsh, Christopher A. | man | Neuron | | 2012 | 29.2 | 146 | 4.5 | 76 | |
| Jessen, Kristjan R. | man | Neuron | | 2012 | 29.0 | 145 | 2.8 | 14 | |
| Fuks, Francois | man | EMBO Journal | | 2012 | 28.6 | 143 | 3.1 | 27 | |
| Jagust, William J. | man | Annals of Neurology | | 2012 | 28.0 | 140 | 7.8 | 144 | |
| Horn, Janneke | woman | Annals of Neurology | | 2012 | 27.8 | 139 | 4.5 | 98 | |
| Chang, Edward F. | man | PLoS Biology | | 2012 | 27.4 | 137 | 3.3 | 101 | |
| Chen, Tsai-Wen | man | Journal of Neuroscience | | 2012 | 27.4 | 137 | 9.2 | 13 | |
| Schrag, Anette | woman | Annals of Neurology | | 2012 | 27.2 | 136 | 2.8 | 46 | |
| de Jonghe, Peter | man | Annals of Neurology | | 2012 | 26.6 | 133 | 3.2 | 81 | |
| Jirsa, Viktor K. | man | Journal of Neuroscience | | 2012 | 26.6 | 133 | 1.8 | 64 | |
| Schapira, Anthony H. V. | man | Annals of Neurology | | 2012 | 26.6 | 133 | 4.4 | 103 | |
| Araque, Alfonso | man | PLoS Biology | | 2012 | 26.4 | 132 | 3.4 | 18 | |
| Yockteng, Roxana | woman | PLoS Biology | | 2012 | 26.4 | 132 | 1.6 | 11 | |

*FWCI: field-weighted citation impact; TC: total citation; wTC; weighted total citation*
